# Controlling for activity‐dependent genes and behavioral states is critical for determining brain relationships within and across species

**DOI:** 10.1002/cne.25157

**Published:** 2021-05-04

**Authors:** Matthew T. Biegler, Lindsey J. Cantin, Danielle L. Scarano, Erich D. Jarvis

**Affiliations:** ^1^ Department of Neurobiology Duke University Medical Center Durham North Carolina USA; ^2^ Laboratory of Neurogenetics of Language The Rockefeller University New York New York USA; ^3^ Howard Hughes Medical Institute Chevy Chase Maryland USA

**Keywords:** comparative anatomy, immediate‐early genes, neuroanatomy, nuclear receptor subfamily 4 group a member 2, songbirds

## Abstract

The genetic profile of vertebrate pallia has long driven debate on homology across distantly related clades. Based on an expression profile of the orphan nuclear receptor *NR4A2* in mouse and chicken brains, Puelles et al. (The Journal of Comparative Neurology, 2016, 524, 665–703) concluded that the avian lateral mesopallium is homologous to the mammalian claustrum, and the medial mesopallium homologous to the insula cortex. They argued that their findings contradict conclusions by Jarvis et al. (The Journal of Comparative Neurology, 2013, 521, 3614–3665) and Chen et al. (The Journal of Comparative Neurology, 2013, 521, 3666–3701) that the hyperpallium densocellare is instead a mesopallium cell population, and by Suzuki and Hirata (Frontiers in Neuroanatomy, 2014, 8, 783) that the avian mesopallium is homologous to mammalian cortical layers 2/3. Here, we find that *NR4A2* is an activity‐dependent gene and cannot be used to determine brain organization or species relationships without considering behavioral state. Activity‐dependent *NR4A2* expression has been previously demonstrated in the rodent brain, with the highest induction occurring within the claustrum, amygdala, deep and superficial cortical layers, and hippocampus. In the zebra finch, we find that *NR4A2* is constitutively expressed in the arcopallium, but induced in parts of the mesopallium, and in sparse cells within the hyperpallium, depending on animal stimulus or behavioral state. Basal and induced *NR4A2* expression patterns do not discount the previously named avian hyperpallium densocellare as dorsal mesopallium and conflict with proposed homology between the avian mesopallium and mammalian claustrum/insula at the exclusion of other brain regions. Broadly, these findings highlight the importance of controlling for behavioral state and neural activity to genetically define brain cell population relationships within and across species.

## Graphical abstract

In 2016, Puelles et al. sought to determine the relationship of avian and mammalian pallial regions using the expression profile of the NR4A2 nuclear orphan receptor, concluding that the lateral avian mesopallium was most like the mammalian claustrum, not layer 2 of the cortex as found by Suzuki and Hirata (Frontiers in Neuroanatomy, 2014, 8, 783). However, we found that the NR4A2 expression patterns were characteristic of an activity‐dependent gene. Looking at different behavioral contexts in the zebra finch, we found induced expression of NR4A2 in regions across the pallium associated with visual, somatosensory, and motor activity, confounding and contradicting previous findings. Accordingly, we do not believe NR4A2 is a suitable gene for assessing homologous brain anatomy without understanding the animal's behavioral state.

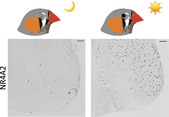

## 1 INTRODUCTION

Gene expression profiling of brain regions and cell types have been used as evidence for determining brain region relationships within and across species. One debate using gene expression has been on homologies of cell populations in the pallium across vertebrate species. In search of ventricle subplate cell marker genes that give rise to the mammalian cortical layers and the homologous pallium across vertebrates, Wang et al. (2011) identified the Nuclear Receptor Subfamily 4 Group A Member 2 (*NR4A2*, also called nuclear receptor related 1, *Nurr1*). They found high expression levels in embryonic and adult cortical subplate and claustrum cells of mammals, and in the hyperpallium on the dorsal surface of the pallium of chickens. They suggested that the mammalian cortical plate neurons could be homologous to avian hyperpallium neurons. Based on these findings and that of Watakabe, Ohsawa, Ichinohe, Rockland, and Yamamori (2014) on *NR4A2* in the claustrum of mammals, Puelles (2014) and Puelles et al. (2016) further studied *NR4A2* in mouse and chicken embryos, and early chicken hatchlings. The Puelles et al. studies reached several major conclusions, including:

1. In mammals, the *NR4A2*‐positive claustrum develops first, followed by some of the *NR4A2*‐positive cells migrating into the insula.
2. In birds, the *NR4A2*‐postive lateral mesopallium is similar to the mammalian claustrum and the medial mesopallium is similar to the mammalian insula, in an outside‐in pattern that is opposite to the inside‐out pattern of mammals.
3. That lateral part of the avian mesopallium (M), similar to the mammalian claustrum relative to the surrounding brain regions, extends into the avian hyperpallium (H) dorsal to it and nidopallium (N) ventral to it.
4. That the avian *NR4A2* pattern in birds contradicts the Jarvis et al., 2013 and Chen et al. (2013) mirror image hypothesis of avian brain organization, where the latter two publications renamed the avian hyperpallium densocellare (HD) as dorsal mesopallium (MD).
5. That the *NR4A2* patterns in birds compared to mice are inconsistent with the Suzuki & Hirata, (Suzuki & Hirata, 2014) hypothesis that the avian mesopallium is homologous to mammalian cortical layers 2/3.

In follow‐up studies and reviews, Watson and Puelles (2017) used the *NR4A2* expression pattern to revise their understanding of the relationship of the claustrum to the endopiriform nucleus ventral to it. This included a newly proposed tetrapartite breakdown of the vertebrate pallium: for birds, dorsal as hyperpallium, lateral as mesopallium, ventral as nidopallium, and medial as hippocampus. This view has been debated (Atoji, Sarkar, & Wild, 2018; Puelles, 2017; Wullimann, 2017a, 2017b), arguing for a different revision of the tetrapartite organization. Wullimann, 2017a suggests a tetrapartite hypothesis for birds of dorsal as hyperpallium and mesopallium, ventral and lateral as nidopallium, and medial as hippocampus. Wullimann took into consideration the combined findings of Watson and Puelles (2017), Jarvis et al. (2013) and Chen et al. (2013). The latter two studies argued that the gene expression evidence does not support a tetrapartite organization of the avian brain. Puelles (2017) further claimed that the patterns of *NR4A2* and another gene, *CYP26B*, showed that the lateral most edge of the avian mesopallium is strictly the homolog of the mammalian claustrum proper, while the rest of the mesopallium is a field homolog of a combination of the mammalian claustrum and insula cortex. These findings were claimed to further justify support for the tetrapartite organization.

A more recent collaboration between the Puelles and Molnar groups (Bruguier et al., 2020) examined *NR4A2* alongside many other genes (40–50 per brain region) from the Allen Institute mouse developmental and adult gene expression Brain Atlases (Lein et al., 2007; Thompson et al., 2014). They found that most claustrum enriched genes were also enriched in cortex layer 6b, but the converse was not found for a number of genes. Further, their preliminary cell lineage tracing experiments found that the newly dividing cells that enter the claustrum or insula from the lateral pallium stay within their respective subdivisions, instead of migrating between these two structures or into layer 6b. Similarly, cells from the avian lateral ventral mesopallium stay within the ventral mesopallium (MV), without migrating dorsally into the lateral hyperpallium or ventrally into the lateral nidopallium. These latter findings did not validate the cell migration hypothesis from the mammalian claustrum or avian mesopallium.

When examining the images in Puelles et al. (2016) and other similar past studies, we noted that the patterns of the *NR4A2* expression appeared quite varied and did not fill entire telencephalic subdivisions, unlike most constitutively expressed genes (Jarvis et al., 2013). Instead, only parts of the mesopallium and hyperpallium were labeled, particularly in late developmental stages and in adults. Puelles (2017) noted that some patterns disappeared in adults. To us, the patterns appeared reminiscent of immediate early genes (IEGs), which are activated in specific cell types of brain circuits dependent on the behavior performed or sensory stimulus processed (Feenders et al., 2008; Jarvis et al., 2013; Jarvis & Nottebohm, 1997).

A literature analysis reveals that *NR4A2* does undergo activity‐dependent expression in certain brain cell types. Most strikingly, robust *NR4A2* expression induction was observed in the adult rat claustrum, deep cortical layers, in some superficial layers, and the hippocampus from 1 to 8 hr following a single subcutaneous injection of kainic acid, which induces seizure activity (Crispino, Tocco, Feldman, Herschman, & Baudry, 1998, particularly their figure 7). We noted that this induced expression pattern recapitulates much of the mouse *NR4A2* expression seen in Puelles (2014) and Puelles et al. (2016). *NR4A2* is part of an orphan nuclear receptor family with noted involvement in NMDAR activity‐mediated and CREB‐dependent survival of granule cells in the rat cerebellum (Barneda‐Zahonero et al., 2012; Volakakis et al., 2010). In cultured mouse hippocampal neurons, *NR4A2* expression is blocked by voltage‐dependent calcium channel inhibition (Tokuoka et al., 2014), indicative of activity‐dependent expression. *NR4A2* has a delayed expression response, as determined by qPCR and microarray assays from rat neurons, compared to its *NR4A3* paralog (Saha et al., 2011). In the zebra finch, a songbird species, the *NR4A1* and *NR4A3* paralogs show a rapid increase in expression in several song nuclei following singing activity (Whitney et al., 2014). Thus, the prior studies on comparative neurobiology (e.g., Puelles, 2014; Puelles et al., 2016) did not take into consideration that the *NR4A2* patterns could be activity‐ or behavioral context‐dependent. This prompted us to look into the *NR4A2* brain gene expression further, using approaches we developed and used to study activity‐dependent gene expression in the avian brain (Feenders et al., 2008; Jarvis & Nottebohm, 1997; Mello & Jarvis, 2008; Whitney et al., 2014).

We found both a basal and stimulus‐/behavior‐driven pattern of *NR4A2* expression in the avian brain, confounding prior hypotheses on avian brain organization and homologies with mammals. Importantly, our findings contradict the interpretations presented in Puelles et al., 2016, do not contradict the renaming of HD as MD nor other aspects of the hypothesis that the dorsal and ventral pallial populations in the avian brain, are similar, and therefore, differing from the tetrapartite hypothesis.

## 2 MATERIALS AND METHODS

### 2.1 Behavioral context and sample collection

Animals were cared for in accordance with the standards set by the American Association of Laboratory Animal Care and Rockefeller University's Animal Use and Care Committee. All animals used for data analysis were collected at Rockefeller University. A total of 12 adult zebra finch males (>90 days old) were used, four for each group described below. Following protocols we developed to measure expression of activity‐dependent genes in the avian brain (Feenders et al., 2008; Jarvis et al., 2013; Whitney et al., 2014), animals were placed individually in sound attenuation chambers overnight (at least 12 hr) to reduce stimulus‐ and behaviorally regulated gene expression to baseline levels, and then treated under the following three conditions:

- *Silent in darkness*: Animals taken prior to the lights turning on in the morning.
- *Silent in light*: Animals were taken after 1.5–2.5 hr of the lights turning on in the morning, moving around, feeding, and drinking, but not singing.
- *Singing*: Animals were monitored and those that produced at least 25 undirected song bouts (continuous ~4–20s periods of songs separated by <500 ms) per 30 min, within 1–1.5 hr after the lights turned on, were taken for the study.

After each condition was complete, animals were quickly euthanized (<1 min) by rapid decapitation, and whole brains were excised, cut mid‐sagittally; separated hemispheres were embedded in block molds containing Tissue‐Tek (Fisher HealthCare, Houston, TX) and quickly frozen in a slurry of dry ice powder and 100% ethanol. The amount of time between removing the bird from the sound attenuation chamber and freezing the brain tissue was under five min, so as to not measure induced gene expression due to the stress of euthanasia. Sections were cut on a CM1950 cryostat (Leica Biosystems, Buffalo Grove, IL) at 12 μm thickness in sagittal or coronal planes, and mounted onto Superfrost Plus slides (Fisher Scientific, Pittsburgh, PA).

### 2.2 Single label in situ hybridization

Plasmids containing RNA polymerase promoters and cDNA sequences for *NR4A2* or other genes of interest were used to amplify the cDNA inserts by PCR. We also used an alternative method, where we isolated the cDNA insert from the plasmid with *PvuII‐HF* (New England Biolabs, Cat. #R3151) or *BssHII* (New England Biolabs, Cat. #R0199) restriction enzymes, targeting restriction sites flanking the probe sequence. The cDNA products were purified using the Nucleospin® Gel and PCR Cleanup kit (Takara Bio, Cat. #740609). The cDNAs were transcribed and labeled following the instructions provided with the DIG RNA Labeling mix or the Fluorescein RNA Labeling mix (Sigma, Cat. #11685619910). The generated RNA probes were purified by ethanol precipitation, resuspended in 90% formamide, and stored at −80 C until further use.

**TABLE 1:** RNA riboprobes used

Gene	Accession number	Antisense RNA polymerase	Sense RNA polymerase	Label
*NR4A2*	CK305076	T3	T7	DIG
*ZENK*	JX296528	T7	SP6	FITC
*ER81*	DV582566	T3	T7	FITC
*FOXP1*	AY549152	T3	T7	FITC

*Note:* Accession numbers correspond to GenBank IDs, available through the National Center for Biotechnology Information (NCBI).

All steps were performed at room temperature (RT) unless specifically noted. Slides containing 4–6 brain sections of a series were fixed with 4% Paraformaldehyde (PFA) in 1× PBS, washed with 1× PBS and then incubated in acetylation buffer (250 ml 0.1 M Triethanolamine, 280 μl NaOH and 625 μl acetic anhydride, mixed right before use). The sections were washed with 1× PBS and dehydrated serially with 70%, 95% and 100% EtOH. Samples were incubated for at least 1 hr in prehybridization solution (50% formamide, 5× SSC, 1× Denhardt's solution, 250 μg/ml tRNA, 500 μg/ml herring sperm DNA).

To hybridize, RNA probes were diluted 1:100 in the hybridization solution (50% formamide, 300 mM NaCl, 20 mM Tris–HCl [pH 8.0], 5 mM EDTA, 10 mM NA_2_HPO_4_, 1× Denhardt's, 500 μg/ml tRNA, 200 μg/ml herring sperm DNA and 10% dextran sulfate). The diluted probe mix was incubated at 80°C for 6 min and then cooled on ice for 5 min. After removing the prehybridization solution, 100 μl of the probe mix was added to each slide. The slides were coverslipped, with care taken not to introduce bubbles, and incubated overnight at 65°C in a HybEZ II hybridization oven (ACD).

Coverslips were removed in 2× SSPE, washed with 1× SSPE/50% formamide for 1 hr at 65°C, and 0.1× SSPE twice for 30 min at 65°C. The slides were cooled to room temperature in the last 0.1× SSPE wash and then incubated in a new 0.1× SSPE wash for 5 min at room temperature. The sections were washed with 1× Tris Buffered Saline (TBS) for 5 min and then incubated for 1 hr in blocking solution (TBS, 10% sheep serum). The slides were incubated in an antibody solution (TBS, 1% sheep serum and Anti‐DIG‐AP (Sigma, Cat. #11093274910, RRID:AB_514497) or Anti‐FITC‐AP (Sigma, Cat. #11426338910, RRID:AB_514504) at 1:2000) overnight at 4°C.

The slides were washed in 1×TBS three times for 10 min each at room temperature and then equilibrated with 0.1 M Tris–HCl pH 9.5 for 5 min. The sections were then incubated in the NBT/BCIP solution (Sigma, Cat. #11681451001) for 2–24 hr (each probe was optimized based on signal) until they were fully developed. The slides were washed with 1× PBS three times for 5 min each, rinsed in diH_2_O, dehydrated with 100% EtOH and mounted under glass coverslips (ThermoFisher, Cat. #12545M) using ProLong Diamond Antifade mounting solution (Invitrogen, Cat. #P36961).

### 2.3 Double‐label in situ hybridization

To determine overlap of multiple genes in the same cells, we performed double‐label fluorescent in situ hybridization. We followed the same steps as the single label in situ hybridization up until the hybridization step. For double labeling, we diluted the two probes of interest (one conjugated with DIG and one with FITC) into the same tube of hybridization solution at a ratio of 1:100 for each probe. We then followed the same steps as the single label protocol, stopping after the final 0.1× SSPE wash for 5 min at room temperature. At that point, slides were washed with 1× PBS for 5 min, incubated in 10% H_2_O_2_‐1× PBS for 10 min to remove any endogenous peroxidase activity and then washed twice in 1× PBS for 5 min. To label the hybridized *NR4A2* DIG‐conjugated probes, the sections were incubated in 0.5% Roche Blocking Solution (Sigma, Cat. #11096176001) for 1 hr and then incubated with Anti‐DIG‐POD antibody (Sigma, Cat. #11207733910, RRID:AB_514500) at 1:1000 dilution in 0.5% Roche Blocking Solution overnight at 4°C.

The slides were washed twice with 1× PBS for 10 min and then once with 0.1% BSA‐1× PBS for 10 min. The remaining steps all occurred while protecting the slides from natural or ultraviolet light, utilizing opaque horizontal slide boxes or vertical slide mailers. Slides were incubated with 1:100 Cy3‐TSA amplification reagent in 1× Plus Amplification Diluent (Akoya Biosciences, Cat. #NEL741001KT) for 15 min, then washed in 1× PBS for 5 min. The slides were then quenched in 15% H_2_O_2_‐1× PBS for 30 min to remove any remaining peroxidase reactivity and washed twice in 1× PBS for 5 min. Slides were fixed in 4% PFA‐1× PBS for 5 min and washed twice with 1× PBS for 3 min to ensure tissue integrity.

For the second round of antibody staining against the hybridized FITC‐conjugated riboprobe (e.g., *FOXP1*, *ER81* [also called *ETV1*], or *EGR1*), the sections were incubated in 0.5% Roche Blocking Solution for 30 min and then incubated with Anti‐FITC‐POD antibody (Sigma, Cat. #11426346910, RRID:AB_840257) at 1:1000 in 0.5% Roche Blocking Solution for 2 hr at room temperature, or overnight at 4°C. The slides were washed twice with 1× PBS for 10 min and then with 0.1% BSA‐PBS for 10 min. The slides were incubated with 1:100 FITC‐TSA in amplification buffer for 15 min, then washed in 1× PBS for 5 min. The sections were counterstained with DAPI‐1× PBS for 15 min. The slides were washed twice in 1× PBS for 5 min and then rinsed with dH2O. They were then coverslipped with Prolong Diamond Antifade mounting media (Invitrogen) and dried overnight at room temperature, protected from light.

**TABLE 2:** Competing models of brain organization and homology compared to *NR4A2* expression

Avian forebrain region	*NR4A2* expression	Proposed mammalian homolog	
		Puelles et al., 2016	Jarvis et al., 2013
Hyperpallium	Sparsely induced, constitutive in parts	Cortex	Cortical layer 3
Intercalated hyperpallium	Not observed	Cortex	Cortical layer 4
Dorsal mesopallium	Induced	Cortex	Cortical layers 1/2
Ventral mesopallium	Induced	Dorsal claustrum	Cortical layers 1/2
Nidopallium	Induced in parts	Ventral claustrum	Cortical layer 3
Intercalated nidopallium	Not observed	Ventral claustrum	Cortical layer 4
Arcopallium	Constitutive and induced in parts	Amygdala	Cortical layers 5/6 & Claustrum/amygdala
Striatum	Not observed	Striatum	Striatum
Pallidum	Weakly induced	Globus pallidus	Globus pallidus

### 2.4 Gene expression quantification

Images of the brain sections were taken at ×4 magnification on an Olympus BX61 upright microscope (colorimetric single‐labeling) or ×10 magnification on an inverted Zeiss LSM 780 laser scanning confocal microscope (fluorescent double‐labeling) and analyzed in Adobe Photoshop CC (version 22.0.1, RRID:SCR_014199). For quantification, images of the brain sections were desaturated and inverted using photoshop commands, and the signal intensity in brain regions of interest was normalized across samples by dividing by background intensity levels of a control brain region (i.e., striatum) qualitatively observed to be free of *NR4A2* mRNA signal. Quantification was not completely blinded, as the *NR4A2* induction was plainly visible across conditions. The marquee tool was used to select a portion of the brain region of interest, and labeled cells were then automatically selected within the region using the Color Range selection tool to select Highlights. The Color Range parameter (0% fuzziness) was strictly set to the lowest nonselecting Range value in control brain regions (i.e., striatum). The number of selected cells were recorded using the Record Measurements tool. These counts were divided by the area of the selected brain region to obtain the number of labeled cells per mm^2^. Significance between groups was measured using ANOVA, followed by Tukey's HSD test for post‐hoc analysis.

## 3 RESULTS

### 3.1 *NR4A2* brain expression pattern varies across behavioral conditions

**FIGURE 1:**
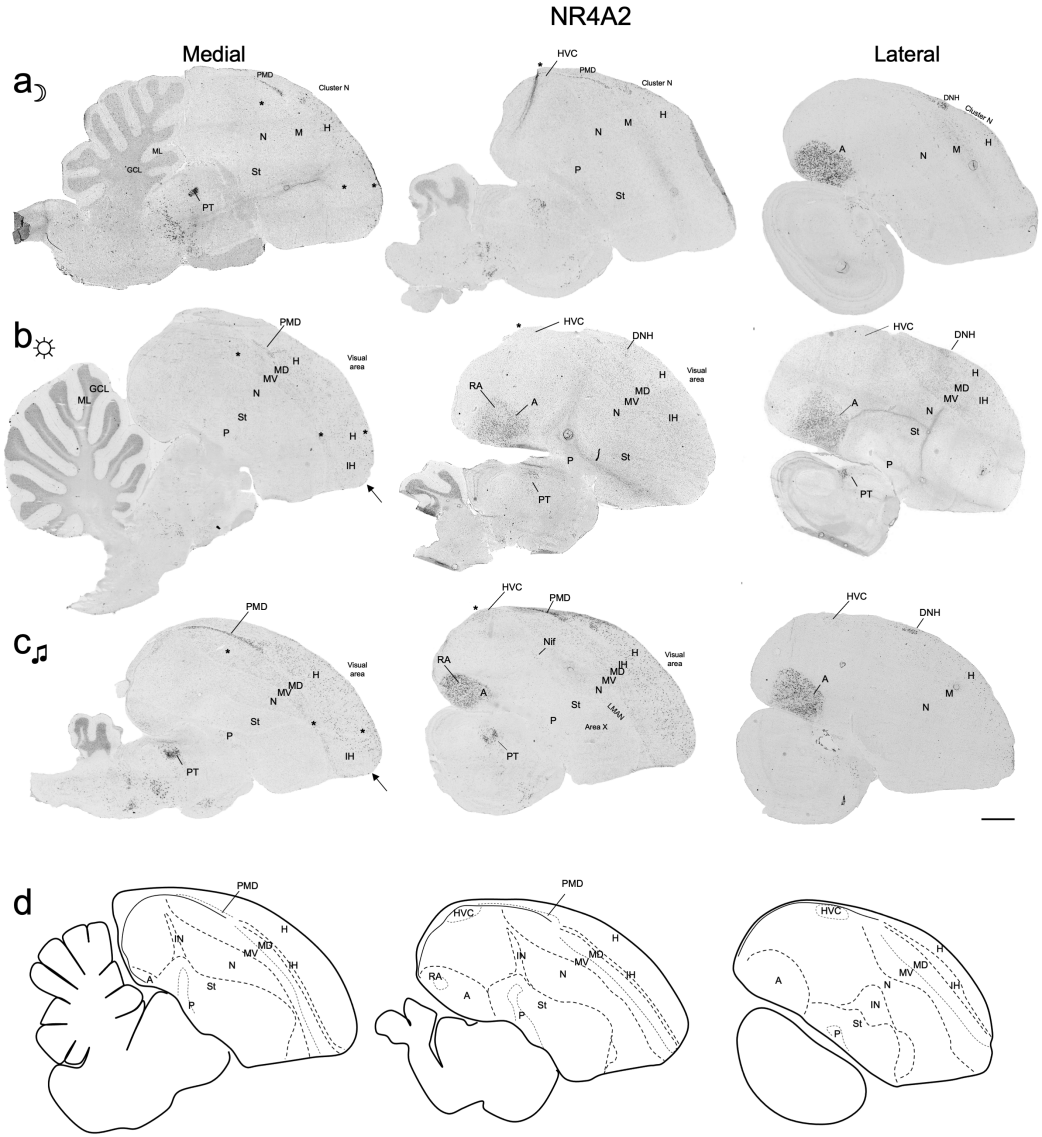
Basal and activity‐dependent induction of *NR4A2* expression in the zebra finch brain. (a) Medial and lateral brain sagittal sections of *NR4A2* in male zebra finches that were in the dark. (b) Sections from an animal exposed to lights for 2 hr, after an overnight in the dark. (c) Sections from an animal exposed to lights and singing at least 50 song bouts within 60 min prior to sacrifice, after overnight in the dark. (d) Illustrations of brain subdivisions based on adjacent Nissl stained and *FOXP1* labeled (Figure S1) sections and equivalent sections of a digital brain atlas (Karten et al., 2013). Note the differences in mesopallium and hyperpallium expression across behavioral conditions. Asterisks denote regions of significant *NR4A2* induction across groups, and arrows denote the somatosensory region of IH. Images are tiled at ×4 magnification, scale bar = 1 mm. Dorsal is up, posterior is left. Abbreviations and corresponding names are shown in abbreviation, Table 1

**FIGURE 2:**
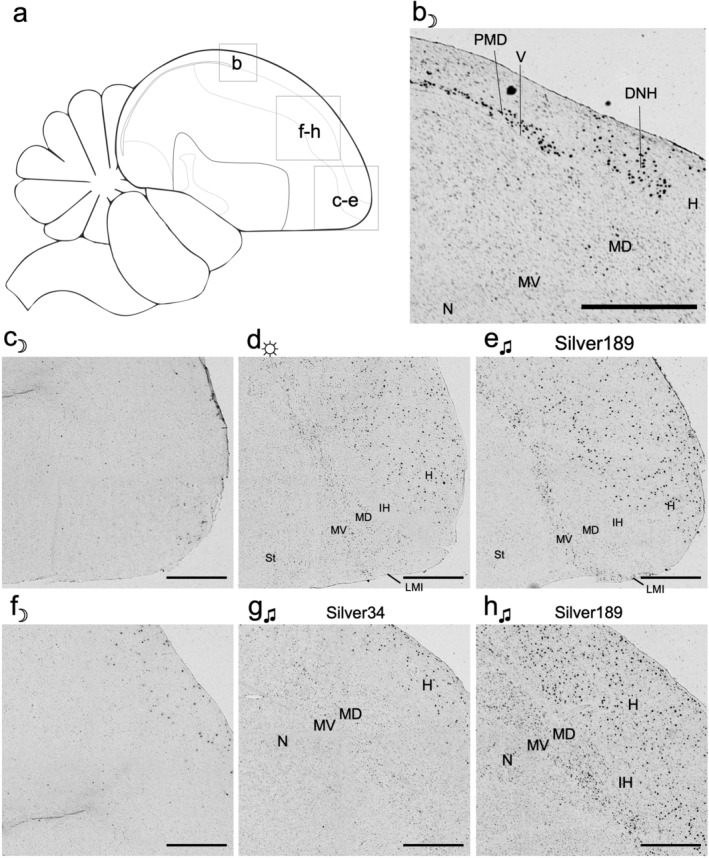
Higher magnification of *NR4A2* brain expression across conditions. (a) Brain diagram of the regions analyzed. (b) *NR4A2* expression in PMD and DNH of a dark‐housed animal. (c–e) Magnified images of the somatosensory regions of hyperpallium, IH, and dorsal mesopallium, from (c) dark, (d) light, and (e) singing animals. (f–h) Magnified images of the visual regions of hyperpallium, IH, and dorsal mesopallium, and motor regions of ventral mesopallium and nidopallium, from (f) dark and (g, h) two singing animals, to show intragroup diversity of expression. Dorsal is up and left is posterior. Images are tiled at ×4 magnification, scale bars = 200 μm. Abbreviations and corresponding names are shown in abbreviation, Table 1

In the dark housed zebra finches, there was high basal *NR4A2* expression in the arcopallium (A), and in the dorsal nucleus of the hyperpallium (DNH; as seen in sagittal sections; Figure 1a). DNH is a brain region involved in night vision and magnetic field sensing in dim light conditions (Mouritsen, Feenders, Liedvogel, Wada, & Jarvis, 2005; Zapka, Heyers, Liedvogel, Jarvis, & Mouritsen, 2010). There was also consistently high expression in a layer of cells directly above the ventricle and posterior to DNH (Figures 1a and 2a), which has been considered either as part of the hippocampus (Shimizu, Bowers, Budzynski, Kahn, & Bingman, 2004; Smulders, Sasson, & Devoogd, 1995) or a posterior extension of the dorsal mesopallium (PMD; Jarvis et al., 2013). There was sparse cell labeling in the hyperpallium around DNH and adjacent MD (terminology as defined in Jarvis et al., 2013, and Chen et al., 2013) overlapping with Cluster N (Figure 1a), a cluster of regions involved in night vision and magnetic field senstation (Mouritsen et al., 2005; Zapka et al., 2010).

**FIGURE 3:**
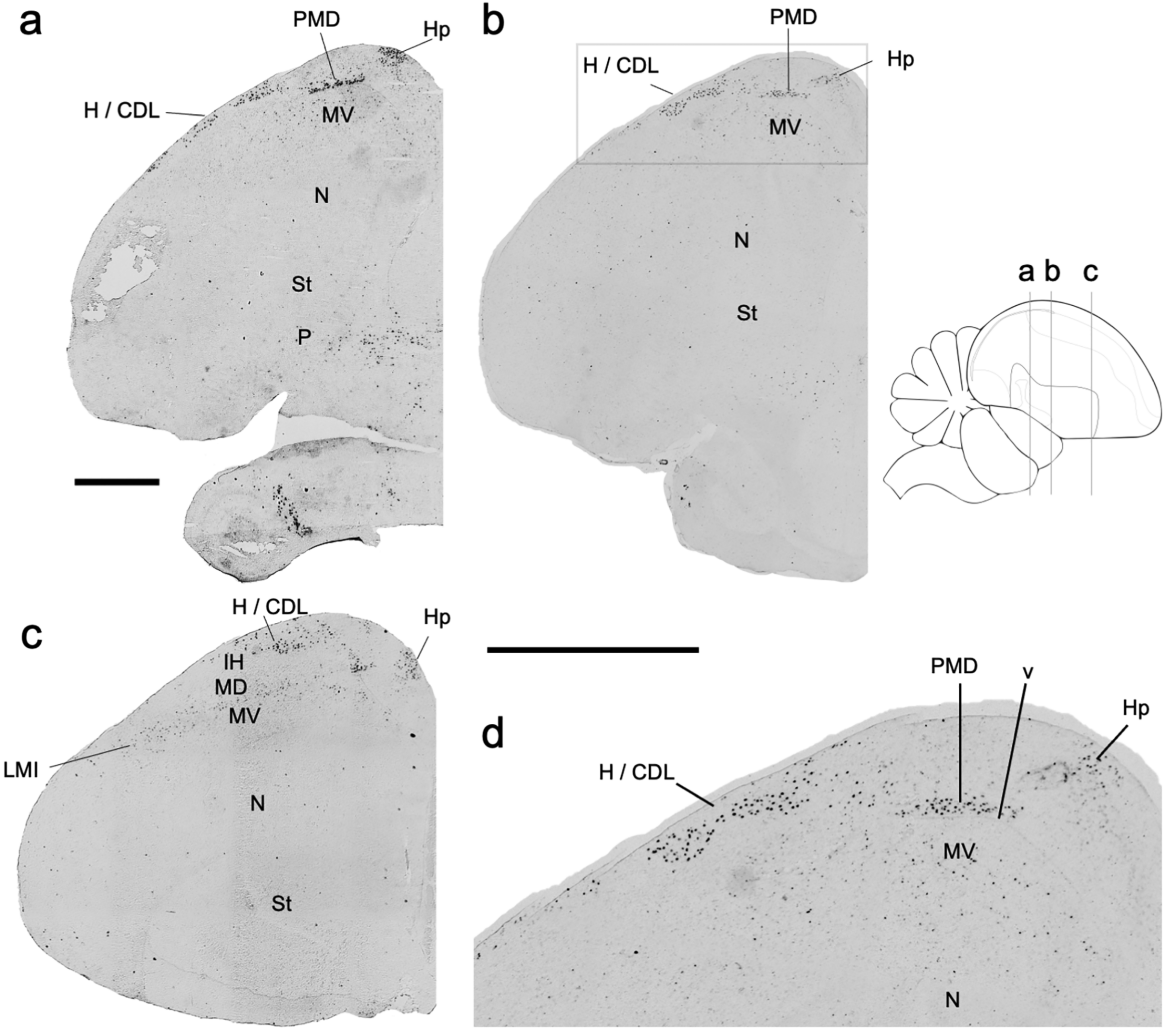
Coronal sections of baseline *NR4A2* expression in the CDL region. (a–c) Coronal sections of a dark housed animal, arranged from posterior to anterior sections. (d) Magnified image of (b) with MD, MV, H/CDL, and PMD highlighted. Images are tiled at ×4 magnification, scale bars = 1 mm. Dorsal is up and lateral is left. Abbreviations and corresponding names are shown in abbreviation, Table 1

There were also strongly labeled cells lateral to the PMD and DNH, near the surface of the brain that connects the hyperpallium with the nidopallium, also called the dorsolateral corticoid area (CDL; best seen in coronal sections, Figure 3a–d). This lateral pallium label varied from one section to another but was seen in all dark housed animals (*n* = 4), corresponding to what Puelles et al. (2016) and Puelles (2017) strictly called the avian claustrum, as a part of lateral mesopallium. However, the cells appeared to be restricted to the hyperpallium (H, as defined in Jarvis et al., 2013). There was low‐level labeling along the boundary lamina (LMI) between the MD and ventral mesopallium (MV; Figure 3c), and some isolated cells in the MV (Figure 3a,b,d), but by no means like the CDL labeling. The granule cell layer of the cerebellum (GCL) also consistently expressed *NR4A2*. In the brainstem, the pretectal (PT) visual nucleus and other nuclei showed strong labeling (Figure 1a). In all remaining telencephalic regions, *NR4A2* expression was very low or undetectable.

**FIGURE 4:**
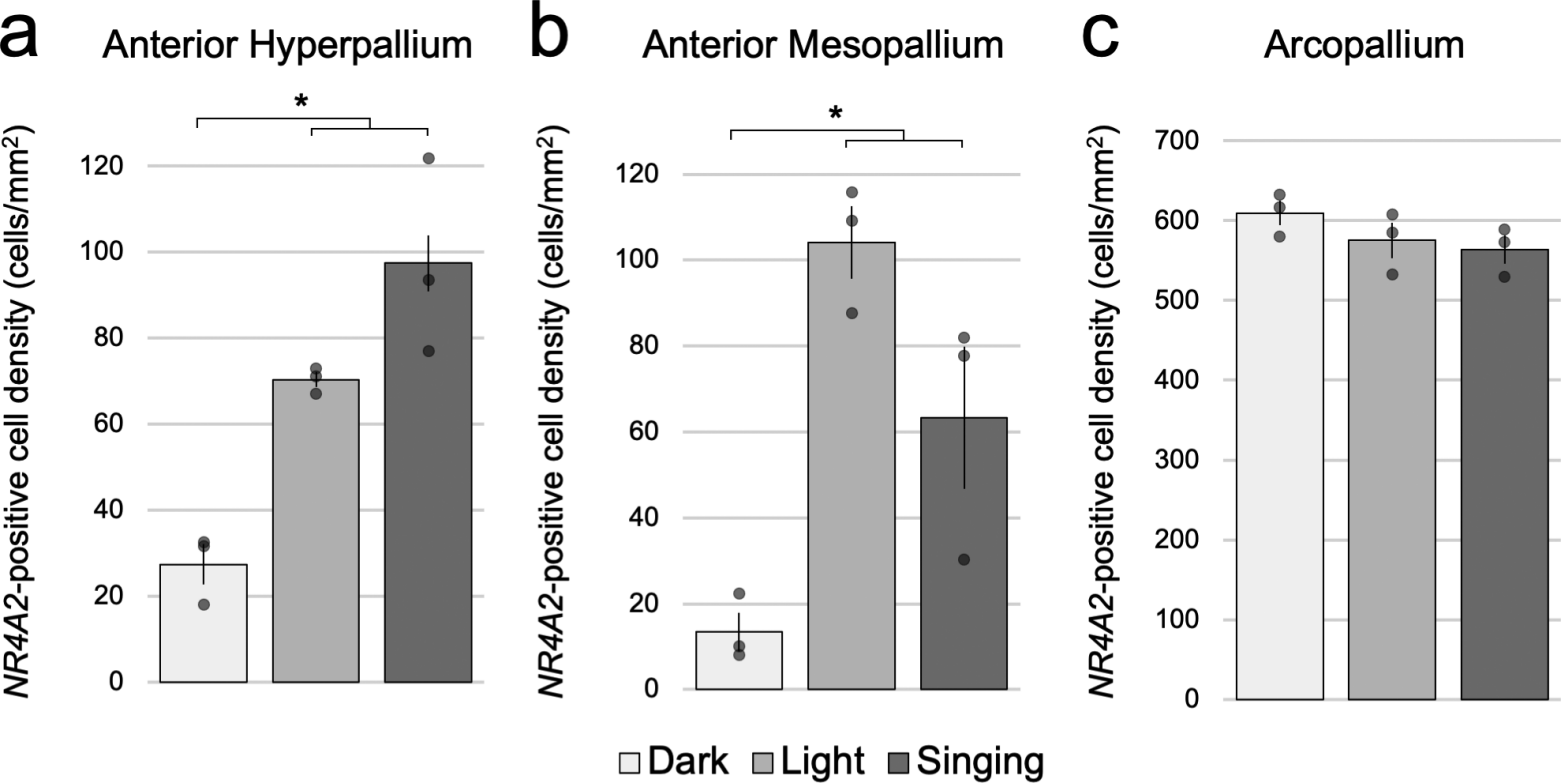
Quantification of activity‐induced *NR4A2* expression. (a–c) Quantification of *NR4A2*‐positive cells/mm^2^ of dark‐housed (*n* = 3), light‐exposed silent (*n* = 3), and singing (*n* = 3) animals in the (a) anterior hyperpallium, (b) anterior mesopallium and (c) arcopallium. Shown are bar graphs, where dots represent values of individual birds, and error bars represent *SEM*. *denotes significance using a one‐factor (behavioral context) ANOVA and Tukey's HSD post‐hoc test, *p* < .05

In light‐exposed animals, in addition to the expression pattern seen in dark housed animals, we noted induced *NR4A2* expression in more isolated cells of the visual hyperpallium (Figure 1b) and anterior somatosensory hyperpallium (Figure 2b,c), as well as adjacent parts of the MD. We noted expression remained within Cluster N (consisting of adjacent parts of H and MD), consistent with the long decay period of *NR4A2* observed in mammals (Crispino et al., 1998; Saha et al., 2011). The induced expression in the somatosensory anterior hyperpallium and adjacent anterior mesopallium was statistically confirmed in quantitative analyses of the number of labeled cells/mm^2^ compared to dark‐housed animals (Figure 4a,b). Induction was found in both the MV and MD (previously named HD, Reiner, Perkel, Mello, & Jarvis, 2004) regions of the mesopallium. There was no quantitative difference seen in the intermediate arcopallium (Figure 4c). Even with these induced levels, the density of *NR4A2*‐positive cells in the arcopallium were still higher than the induced expression in the hyperpallium and mesopallium.

**FIGURE 5:**
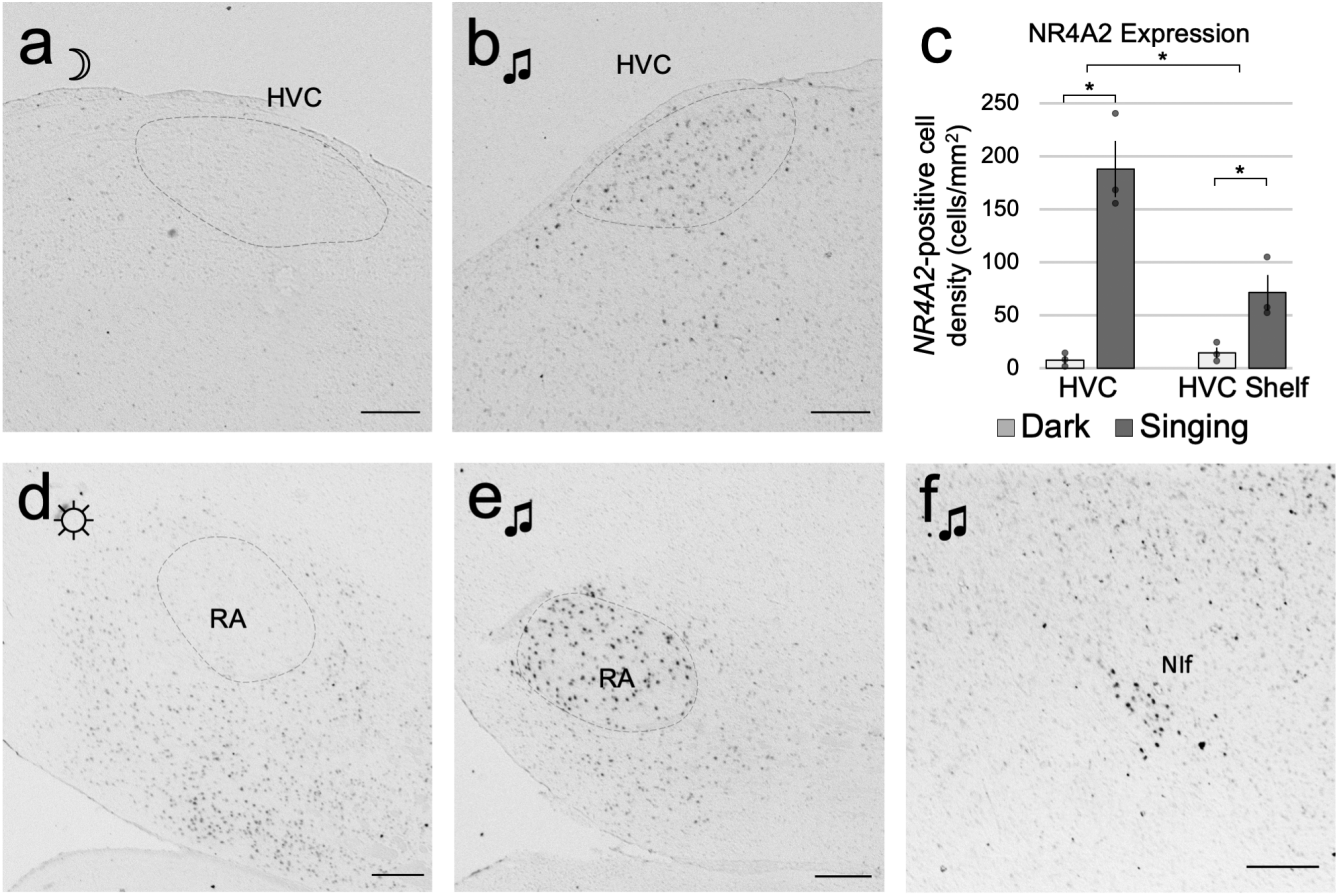
Song‐induced *NR4A2* expression in the song system. (a, b) *NR4A2* nidopallial song nucleus HVC in (a) dark silent and (b) light singing animals. (c) Quantification of *NR4A2* expression in HVC and HVC shelf, by cells/mm^2^, of dark silent (*n* = 3) and singing groups (*n* = 3). Shown are bar graphs, where dots represent values of individual birds, and error bars represent *SEM*. (d, e) The arcopallial song nucleus RA in (d) light silent and (e) light singing animals at low chromogenic exposure conditions. (f) Singing‐driven expression in NIf. Scale bars = 200 μm. * denotes significance using a two‐factor (behavioral context; region) ANOVA and Tukey's HSD post‐hoc test, *p* < .05

In the singing animals, in addition to the patterns seen in the light housed animals (Figures 2b–g and 4a–c), induced *NR4A2* expression was most notably seen in the HVC song nucleus (Figures 1c and 5a,b). Quantitative analysis revealed a 25‐fold increase in mean HVC expression over dark‐housed animals (Figure 5c). Singing‐induced expression was also observed in the RA song nucleus (Figure 5d, e). This difference was best revealed in shorter incubation times of the chromogenic reactions, as longer exposure times needed for other pallial regions saturated the arcopallium and obscured differential expression signals in RA. In an animal that sang the most (Silver189; ~104 song bouts), we also saw increased *NR4A2* expression in the NIf song nucleus (Figures 1c and 5f) but not in the LMAN song nucleus, even though both are located in the nidopallium. We noted overall low expression throughout other parts of the nidopallium in all animals, but even when present at low levels, it appeared to be adjacent to a region of higher expression in MV (Figure 2h), reminiscent of the columnar activation for other IEGs reported in Jarvis et al. (2013). We did not observe *NR4A2* expression in Area X (Figure 1c) or any other striatal region across all behavioral cohorts, indicating that *NR4A2* is not expressed in the avian striatum, similar to previous observations in mammals (Crispino et al., 1998; Puelles, 2014).

In both light‐exposed and singing animals, we noted large variability in the expression pattern from animal to animal in the hyperpallium and the mesopallium. Some animals demonstrated patchy expression, while other animals had continuous expression across the entire subdivision in some sections (Figure 2f vs. g). Compared to the silent in light animals, the mesopallial expression trended lower in the singing animals, due at least in part to higher variation between animals (Figure 4b). The variability of the patterns from animal to animal is indicative of activity‐dependent gene expression, presumably due to differences in behavior and processed stimuli (Jarvis et al., 2013).

**FIGURE 6:**
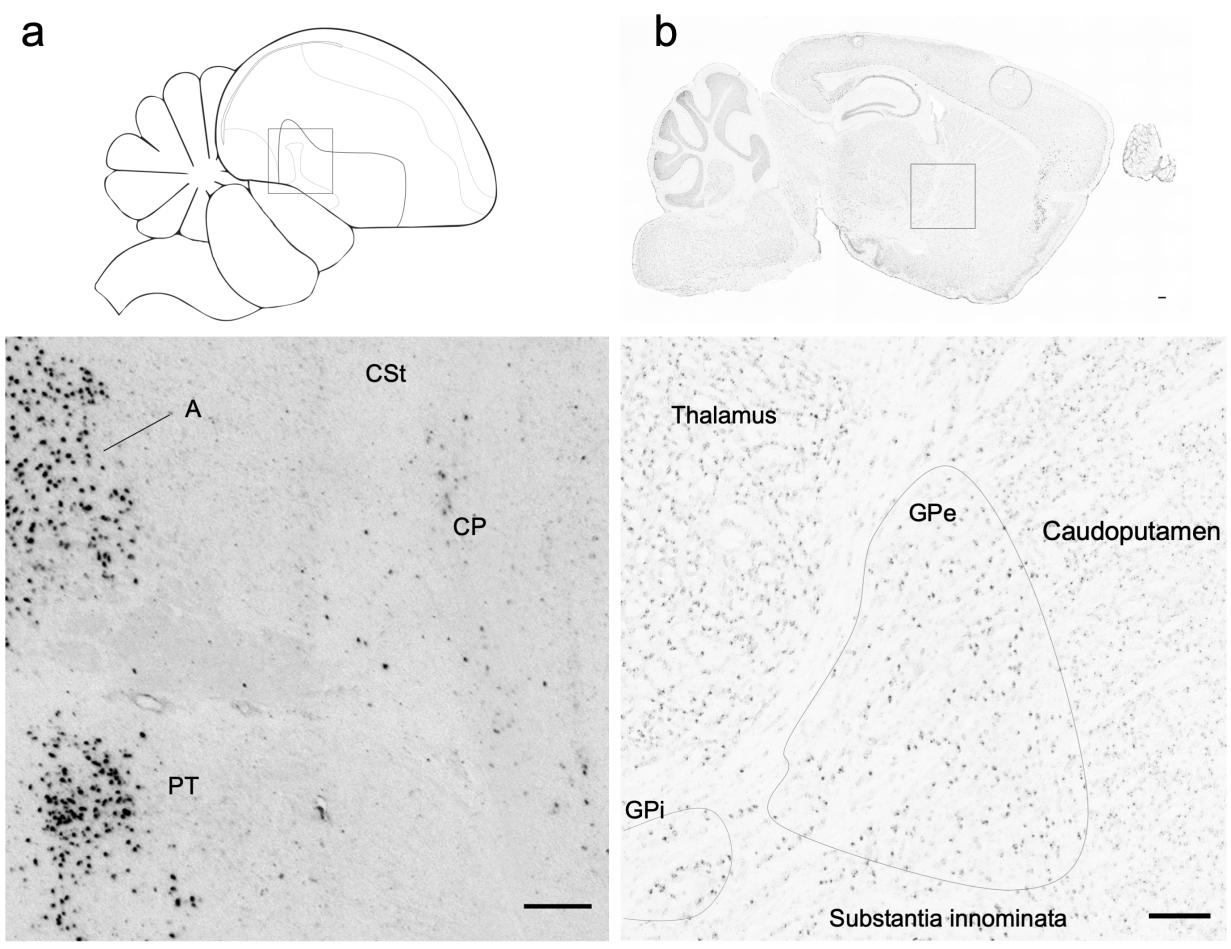
*NR4A2* expression in the pallidum. (a) Noncortical regions of high *NR4A2* expression, including the thalamic pretectal nucleus (PT) and sparse, low‐level induction in the caudal pallidum (CP) of a singing animal, Silver189. The arcopallium (A) is included for reference. (b) Whole brain (top) and (bottom) magnified image of the mouse internal globus pallidus (GPi) and external globus pallidus (GPe), showing low but elevated *NR4A2* expression compared to the adjacent striatum (caudoputamen) in an uncontrolled behavioral context. Scale bars = 200 μm. Image credit for mouse in situ hybridization: Allen Institute for Brain Science, available from http://mouse.brain‐map.org/experiment/show/733

In some singing animals, we noted sparse, low‐level induction of *NR4A2* in the caudal pallidum (CP; Figure 6a), which is directly ventral to the auditory part of the caudal striatum (CSt) that shows activity‐dependent gene expression responses when birds hear song (Feenders et al., 2008; Jarvis et al., 2013). We hypothesized that this induced expression in the pallidum may be due in part to the birds hearing themselves sing. Interestingly, in sagittal sections from a behaviorally undocumented mouse from the Allen Brain Atlas labeled with *NR4A2*, we observed very weak expression in the homologous globus pallidus (Figure 6b). This mouse also had high expression in the claustrum and parts of layer 6b of the cortex, confirming these regions in mammals have increased *NR4A2* expression (see Allen Mouse Brain Atlas [RRID:SCR_002978], *NR4A2* experiment 733). Overall, these findings demonstrate that *NR4A2* brain expression in birds can be activity induced, as in mammals. Such induced expression showed selective patterns, consistent with the specific functions of the brain region or circuit subset involved.

### 3.2 Double labeling clarifies brain subdivision boundaries, cell types, and subcircuits activated

**FIGURE 7:**
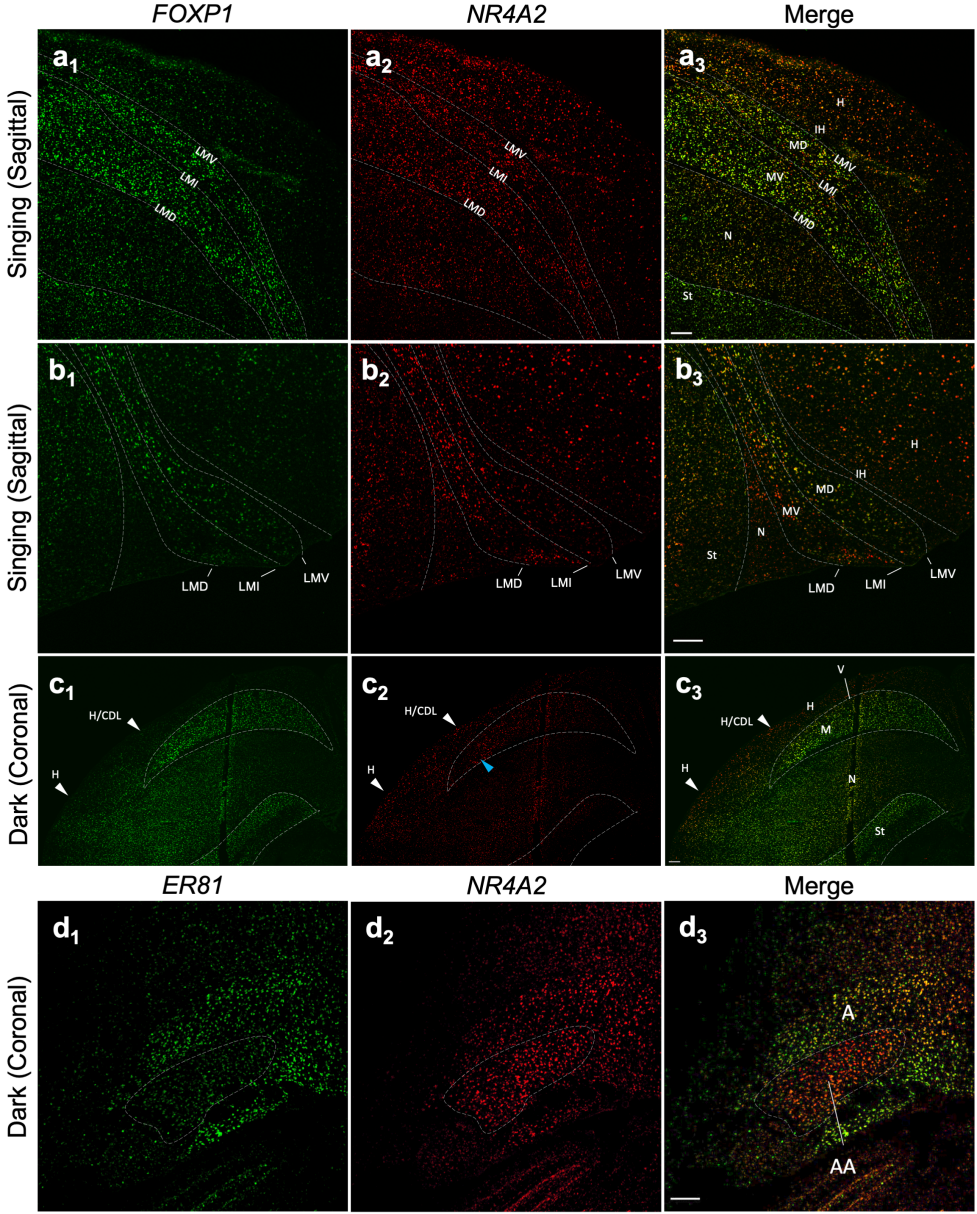
Double labeling of *NR4A2* expression with known pallial markers. (a, b) Sagittal sections of a light stimulated and singing animal with *FOXP1* (FITC‐green) and *NR4A2* (Cy3‐red) labeled in the (a) posterior and (b) anterior mesopallium and hyperpallium, highlighting the difference between *NR4A2* and *FOXP1* across pallial layers. Note the absence of *NR4A2* expression in IH, with the mesopallial boundaries distinguished with *FOXP1*. (c) Coronal section of a dark housed animal co‐labeled with *FOXP1* (FITC‐green) and *NR4A2* (Cy3‐red) highlighting the CDL that connects hyperpallium and nidopallium as separate from the dorsal mesopallium. (d) Coronal section of a dark housed animal co‐labeled with *ER81* (FITC‐green), which labels the arcopallium except its anterior nucleus (AA) and *NR4A2* (Cy3‐red), which labels all of the arcopallium. Images are made from tiled segments under confocal microscopy, scale bars = 200 μm. Dorsal is up, left is medial or posterior in the coronal section and sagittal sections, respectively. Abbreviations and corresponding names are shown in abbreviation, Table 1

With limited basal expression patterns and specific activity‐dependent *NR4A2* expression patterns, it can be difficult to determine the full extent of brain subdivision boundaries when examining it by in situ expression alone. To more concretely verify the locations of *NR4A2* cells in the hyperpallium and mesopallium regions, we performed double labeled in situ hybridization with *FOXP1*, a strong mesopallium marker that distinguishes MV and MD from the hyperpallium and the intercalated hyperpallium (IH) in between them (Figure S1; Jarvis et al., 2013). In the light stimulated and singing animals with the strongest expression, we noted that the *NR4A2* expression was restricted to the MV, MD, and hyperpallium in both posterior (Figure 7a) anterior (Figure 7b) regions. In contrast, the IH was negative for NR4A2 expression (Figure 7a,b; also Figure 2c–g). This *NR4A2* negative pattern in IH is what Puelles et al. (2016) claimed to be HD, which is not our revised MD (Jarvis et al., 2013). We also noted that mesopallial cells with *NR4A2* induction co‐expressed *FOXP1* (Figure 7a,b). The pattern of *NR4A2* seen in the anterior mesopallium and hyperpallium of the active animals is reminiscent of induced IEG expression in the posterior visual and anterior somatosensory parts of these brain regions when animals are very active (Feenders et al., 2008). In coronal sections, the strongly labeled *NR4A2* cells in the CDL were not within the *FOXP1*‐bounded mesopallium, thus confirming their location more dorsally in the hyperpallium (Figure 7c). The LMI border region of higher *NR4A2* expression seen in the chromogenic images (Figure 3c) was also observed within the *FOXP1* boundaries (see blue arrowhead in Figure 7c_2_) with *NR4A2* expression filling portions of both MV and MD.

To verify the *NR4A2*‐positive cells in the arcopallium, we performed double labeling with the *ER81* transcription factor, a marker of avian arcopallial neurons and mammalian layer 5 projection neurons and pallial amygdala (Crispino et al., 1998; Jarvis et al., 2013). Using coronal sections, we found that both genes co‐expressed in many cells of the arcopallium (Figure 7d). One exception was in the anterior arcopallium (AA) nucleus, where *NR4A2* was highly expressed and *ER81* expression was low (Figure 7d). The AA is known to have a distinct gene expression profile from the rest of the arcopallium (Jarvis et al., 2013; Mello, Kaser, Buckner, Wirthlin, & Lovell, 2019).

**FIGURE 8:**
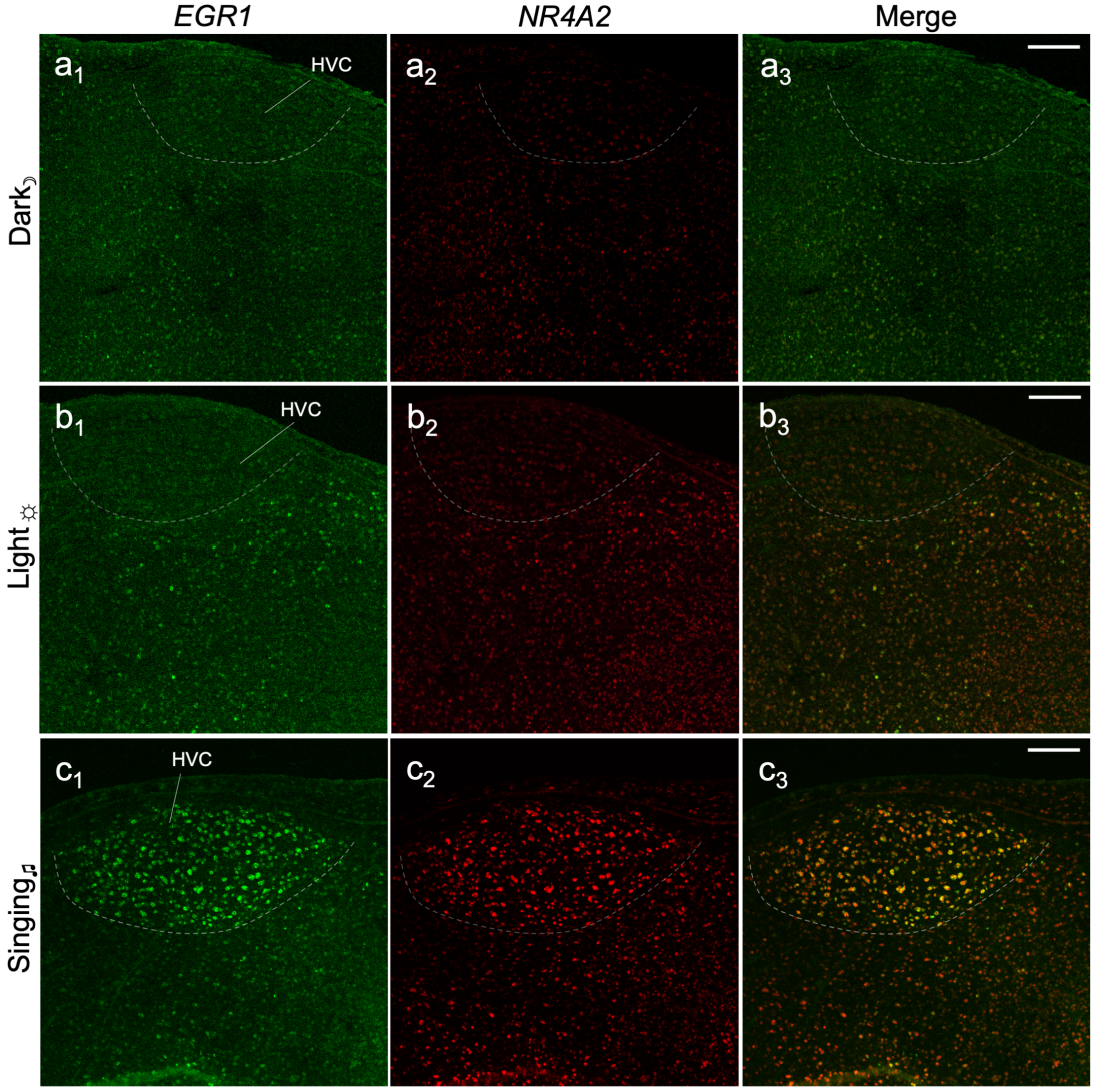
Double labeling of singing‐induced *NR4A2* expression in the song system. Double‐labeled in situ hybridization of *NR4A2* and *EGR1* in HVC highlighted in (a) silent dark, (b) silent light, and (c) singing animals. Dorsal is up, posterior is left. Imaged at ×10 magnification by confocal microscopy, scale bars = 200 μm

For the singing animals, we performed double‐labeling of *NR4A2* with a well‐studied IEG, *EGR1* (Mello & Jarvis, 2008). Compared to nonsinging controls, we found double labeled *NR4A2* + *EGR1* cells throughout HVC (Figure 8a–c) and RA (not shown), whereas only *EGR1* was expressed in the LMAN and Area X song nuclei (not shown). There was also activity‐induced *NR4A2* + *EGR1* expression in the HVC shelf (Figure 8b,c), a nidopallial auditory area. In summary, the double labeling experiments support the hypothesis that a subset of the circuits and brain subdivisions for a particular behavior or stimulus have activity‐induced *NR4A2* expression.

## 4 DISCUSSION

**FIGURE 9:**
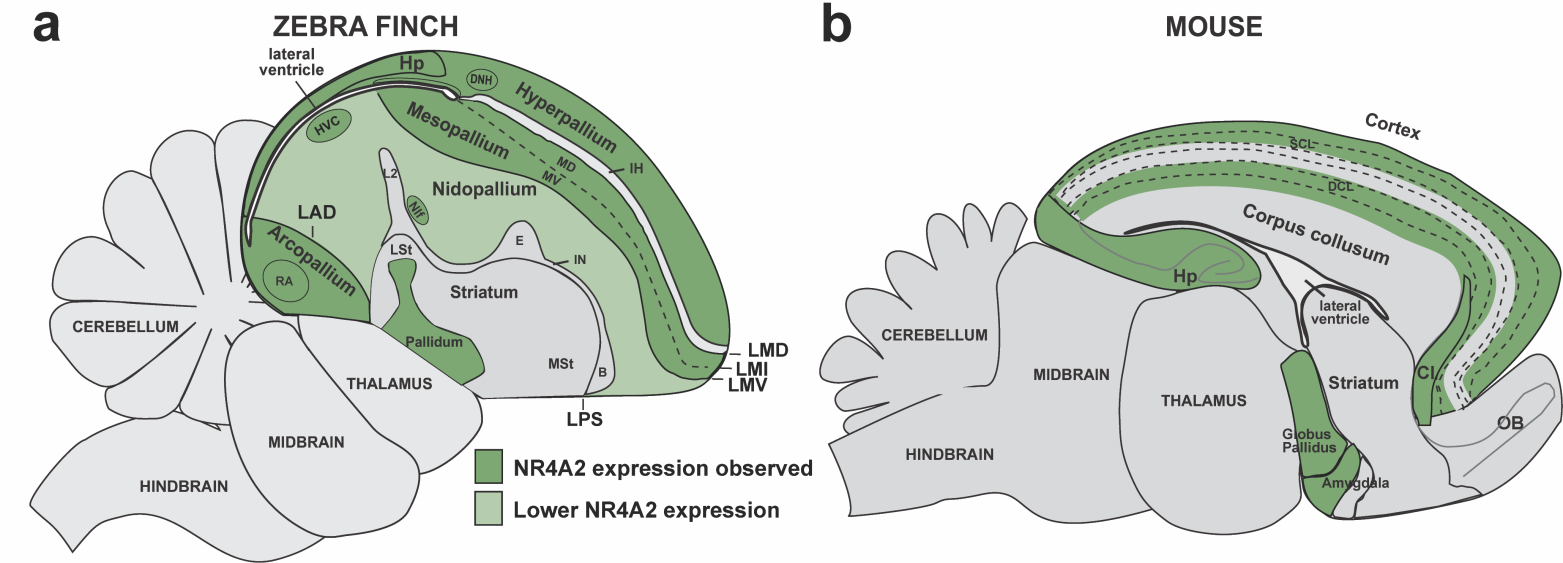
Brain subdivision boundaries of basal and activity‐dependent induction of *NR4A2* expression in the avian and mammalian brains. (a) Model of the extent of brain regions with high (dark green) and low (light green) activity‐induced *NR4A2* expression the avian brain, using the songbird as an example. (b) Model of NR4A2 expression in the mammalian brain, using the mouse as an example. Expression bounds are based on this study, Crispino et al., 1998, Puelles, 2014, Puelles et al., 2016. Further details are in Table 2. Abbreviations and corresponding names are shown in abbreviation, Table 1

Our findings demonstrate that *NR4A2* is an activity‐dependent gene regulated by behavioral and sensory stimuli in the avian brain, as in mammals (Crispino et al., 1998; Saha et al., 2011; Tokuoka et al., 2014). High basal expression in the telencephalon is restricted to the arcopallium and several hyperpallial regions, and high activity‐dependent expression is enriched in the hyperpallium and mesopallium, and less so in the nidopallium outside of the song nucleus HVC (Figure 9a; Table 2, column 2). These clusters of induced expression cross subdivision boundaries in regions that make up circuits for specific behaviors or processing of stimuli (Horita et al., 2012; Horita, Wada, Rivas, Hara, & Jarvis, 2010; Whitney et al., 2014). While we did not investigate the time course of expression, it is possible that robust activity‐dependent induction through longer durations of behavior (Whitney et al., 2014) or systemic kainic acid experimental stimulation may further extend the expression profile of *NR4A2* into areas not seen. However, such kainic acid *NR4A2* induction in the rat has been limited to the claustrum, deep and superficial cortical layers, and hippocampus (Crispino et al., 1998), and this pattern was stable between 1 and 4 hr after induction, consistent with the limited regions of induction we see here in the zebra finch. It is imperative to correctly understand the functional brain organization patterns of this gene, as mutations of human *NR4A2* have been linked to schizophrenia (Buervenich et al., 2000), Parkinson's Disease (Liu et al., 2017), and neuroprotection for Alzheimer's Disease (Moon et al., 2019).

There are two current competing hypotheses on avian pallial organization. The first is the *discontinuum hypothesis* (named as such in our companion study, Gedman et al., 2021), where the dorsal pallium regions above the vestige of the lateral ventricle (collectively called the hyperpallium) are considered distinct from the pallial regions below the ventricle (Table 2, column 3). The second is the *continuum or mirror‐image hypothesis*, where the pallial subdivisions above and below the lateral ventricle are considered three continuous cell populations that wrap around the ventricle (Table 2, column 4). Accordingly, these hypotheses have driven debate between researchers. In the context of the latter hypothesis, Puelles et al., 2016 claim that *“[The] proposal of* *2013* *) and Chen* et al. *2013*.” However, the Jarvis and Chen studies did not state that IH belonged to the mesopallium, but instead that it is a separate cell population from the hyperpallium and mesopallium, with its own molecular profile that receives heavy thalamic input similarly to intercalated nidopallium (IN; Field L2, entopallium, and basorostralis). The sharply negative HD/IH area seen in the Puelles et al., 2016 study is recapitulated here in active adult animals as a sharply negative region between hyperpallium and MD (old HD).

Puelles et al., 2016 (their figure 2) also named the *NR4A2*‐expressing DNH as the intercalated core nucleus (ICo) and postulated it to be “*a migrated claustral derivative*,” though did not follow‐up further. In songbirds, there is clear evidence that DNH is a visual brain region associated with magnetic field sensation (Mouritsen et al., 2005; Zapka et al., 2009). We further advise against naming this structure ICo to avoid confusion with the long‐standing abbreviation of a well‐characterized avian midbrain region, the intercollicular complex (ICo; Wild, Li, & Eagleton, 1997; Kingsbury, Kelly, Schrock, & Goodson, 2011).

The *NR4A2* activity‐dependent induction profile we observed here is more consistent with the continuum hypothesis of cell relationships, as the patterns of expression are comparable in MD and MV on either side of the vestigial ventricle (LMI lamina). One inconsistency with this hypothesis is the weaker induction of *NR4A2* in the nidopallium compared to the hyperpallium. However, the *NR4A2* label in the hyperpallium appears in sparsely labeled cells, which has been seen for only a few other genes (e.g., *SATB2*) that differentially label the hyperpallium relative to the nidopallium, and may point to a unique cell type in the hyperpallium. This is discussed further in our companion study (Gedman et al., 2021). There, we also provide further support for the Jarvis et al. (2013) and Chen et al. (2013) model, as the profiles of nearly all 20,000 annotated genes (including *NR4A2*) align MD (previously HD) to what has been called the mesopallium, and further aligns the overlying IH as most similar to IN.

Do the basal and activity‐dependent expression patterns of *NR4A2* in the avian and mammalian brains support the conclusions of the Puelles studies? Answering this question requires understanding the context of another two competing sets of hypotheses on brain homology between birds and mammals (Jarvis et al., 2005; Jarvis et al., 2013; Reiner et al., 2004). The first of these is the *nuclear‐to‐claustrum‐amygdala hypotheses*, where the avian mesopallium, nidopallium, and arcopallium are considered homologous to parts of the mammalian claustrum and amygdala, while the hyperpallium is homologous to the mammalian six layered cortex (Table 2, column 3). The second set is the *nuclear‐to‐layered cortical hypotheses*, where the avian mesopallium, nidopallium, and arcopallium are instead considered homologous to different layers of the mammalian cortex (Table 2, column 4). As outlined in the introduction, Puelles et al., 2016 suggested a major change to the *nuclear‐to‐claustrum‐amygdala hypotheses* based on the *NR4A2* expression pattern.

However, the basal levels of *NR4A2* in the claustrum and activity‐induction patterns in the adjacent deep cortical layers (Crispino et al., 1998) indicate that this pattern is not consistent with it being homologous to the lateral regions of the avian hyperpallium and adjacent mesopallium. The dorsolateral, *NR4A2*‐labeled region they found in the avian pallium is the CDL region of the hyperpallium, not the lateral mesopallium. The continuous expression of *NR4A2* in the lateral ventral mesopallium and adjacent nidopallium, (CMM and NCM, in some animals) would be consistent with an activity‐induced pattern in functional columns of activation between these two brain subdivisions (Jarvis et al., 2013). The lower *NR4A2* expression seen in the medial mesopallium could be interpreted as not being activated prior to tissue collection. In the Puelles (2014, 2017) and Puelles et al. (2016) studies, there was no clear interpretation mentioned on the relatively low *NR4A2* expression levels in the avian nidopallium, which they previously called the ventral claustrum (Table 2; Puelles et al., 2000). There was also no interpretation offered for the high basal *NR4A2* levels in the avian arcopallium, but not in the proposed mammalian homolog, which they and others designate as the pallial amygdala.

For alternative explanations, one could argue for species differences between chicken (Puelles et al., 2016) and zebra finch (this study), though Wang et al. (2011) analyzed *NR4A2* (*Nurr1*) expression in adult chickens and found labeling mostly restricted to the hyperpallium, without labeling in the mesopallium, reminiscent of patterns we see here in some zebra finches. Some patterns may also be recapitulated across multiple individuals at the same developmental stage, which we believe would more likely demonstrate a stereotypical activity pattern of developing neural circuits (Antón‐Bolaños et al., 2019) than distinct brain subdivision boundaries.

Given that the patterns are not consistent with the homology arguments in the Puelles et al. studies or a tetrapartite avian brain organization, we wonder whether there is support for alternative hypotheses. One could interpret the high *NR4A2* expression patterns in the mammalian deep cortical layers as consistent with homology to the avian arcopallium (Table 2). The weaker induction in the superficial cortical layers of mammals (Crispino et al., 1998) would be consistent with homology to the avian mesopallium and hyperpallium. But these anatomical delineations are based on only one activity‐dependent gene. Two studies, one using in situ hybridization expression profiles of seven critical transcription factors for cortex development (Suzuki & Hirata, 2014) and the other using micro‐array expression profiles of over 7000 orthologous genes (Pfenning et al., 2014), concluded that mammal cortex layers 2 and/or 3 were most molecularly similar to the avian mesopallium or nidopallium, respectively.

There are other claims made in Puelles et al., 2016 and subsequent studies (Puelles, 2017; Watson & Puelles, 2017) using *NR4A2*, which we argue are confounded without proper consideration of the animal's activity state. The importance of controlling animal behavior state and awareness of a gene's activity‐regulated expression have been discussed and demonstrated in past studies, including for birds (Jarvis et al., 2013; Mello & Jarvis, 2008). Interpretations can change dramatically when taking brain activity states into consideration. This is still necessary to consider in embryos despite difficulties in controlling behavior *in ovo*. With our demonstration of differences in interpretation between studies using only this one gene, we hope that future studies will more seriously take behavioral and stimulus context into consideration.

### PEER REVIEW

The peer review history for this article is available at https://publons.com/publon/10.1002/cne.25157.

## Supporting information


**FIGURE S1** Adjacent sections of the (a) dark silent, (b) light silent, and (c) singing animals in Figure 1, hybridized with *FOXP1* to show the boundaries of the mesopallium with the hyperpallium and nidopallium. Tile imaged at ×4 magnification, scale bar = 1 mm. Dorsal is up, posterior is left. Abbreviations and corresponding names are shown in Table 1

## Data Availability

The data supporting the findings of this paper are primarily presented within the scope of this publication. Additional images and materials are available upon request to the corresponding author (EDJ).

## References

[cne25157-bib-0001] Antón‐Bolaños, N. , Sempere‐Ferràndez, A. , Guillamón‐Vivancos, T. , Martini, F. J. , Pérez‐Saiz, L. , Gezelius, H. , et al. (2019). Prenatal activity from thalamic neurons governs the emergence of functional cortical maps in mice. Science, 364(6444), 987–990. 10.1126/science.aav7617 31048552 PMC7611000

[cne25157-bib-0002] Atoji, Y. , Sarkar, S. , & Wild, J. M. (2018). Differential projections of the densocellular and intermediate parts of the hyperpallium in the pigeon (*Columba livia*). Journal of Comparative Neurology, 526(1), 146–165. 10.1002/cne.24328 28891049

[cne25157-bib-0003] Barneda‐Zahonero, B. , Servitja, J.‐M. , Badiola, N. , Miñano‐Molina, A. J. , Fadó, R. , Saura, C. A. , & Rodríguez‐Alvarez, J. (2012). Nurr1 protein is required for N‐methyl‐D‐aspartic acid (NMDA) receptor‐mediated neuronal survival. The Journal of Biological Chemistry, 287(14), 11351–11362. 10.1074/jbc.M111.272427 22294685 PMC3322860

[cne25157-bib-0005] Bruguier, H. , Suarez, R. , Manger, P. , Hoerder‐Suabedissen, A. , Shelton, A. M. , Oliver, D. K. , et al. (2020). In search of common developmental and evolutionary origin of the claustrum and subplate. The Journal of Comparative Neurology, 16(2), 55–22. 10.1002/cne.24922 32266722

[cne25157-bib-0006] Buervenich, S. , Carmine, A. , Arvidsson, M. , Xiang, F. , Zhang, Z. , Sydow, O. , et al. (2000). *NURR1* mutations in cases of schizophrenia and manic‐depressive disorder. American Journal of Medical Genetics, 96(6), 808–813. 10.1002/1096-8628 11121187

[cne25157-bib-0048] Chen, C.‐C. , Winkler, C. M. , Pfenning, A. R. , & Jarvis, E. D. (2013). Molecular profiling of the developing avian telencephalon: Regional timing and brain subdivision continuities. Journal of Comparative Neurology, 521(16), 3666–3701. 10.1002/cne.23406 PMC3863995 23818174

[cne25157-bib-0007] Crispino, M. , Tocco, G. , Feldman, J. D. , Herschman, H. R. , & Baudry, M. (1998). Nurr1 mRNA expression in neonatal and adult rat brain following kainic acid‐induced seizure activity. Brain Research. Molecular Brain Research, 59(2), 178–188. 10.1016/s0169-328x(98)00143-0 9729370

[cne25157-bib-0008] Feenders, G. , Liedvogel, M. , Rivas, M. , Zapka, M. , Horita, H. , Hara, E. , et al. (2008). Molecular mapping of movement‐associated areas in the avian brain: A motor theory for vocal learning origin. PLoS One, 3(3), e1768. 10.1371/journal.pone.0001768 18335043 PMC2258151

[cne25157-bib-0009] Gedman, G., Haase, B., Durieux, G., et al. (2021). As above, so below: Whole transcriptome profiling demonstrates strong molecular similarities between avian dorsal and ventral pallial subdivisions. The Journal of Comparative Neurology. 10.1101/2020.11.13.375055 PMC8251894 33871048

[cne25157-bib-0010] Horita, H. , Wada, K. , Rivas, M. V. , Hara, E. , & Jarvis, E. D. (2010). The dusp1 immediate early gene is regulated by natural stimuli predominantly in sensory input neurons. The Journal of Comparative Neurology, 518(14), 2873–2901. 10.1002/cne.22370 20506480 PMC2904818

[cne25157-bib-0011] Horita, H. , Kobayashi, M. , Liu, W.‐C. , Oka, K. , Jarvis, E. D. , & Wada, K. (2012). Specialized motor‐driven dusp1 expression in the song systems of multiple lineages of vocal learning birds. PLoS One, 7(8), e42173. 10.1371/journal.pone.0042173 22876306 PMC3410896

[cne25157-bib-0012] Jarvis, E. D. , & Nottebohm, F. (1997). Motor‐driven gene expression. Proceedings of the National Academy of Sciences United States of America, 94(8), 4097–4102. 10.1016/0166-2236(91)90027-R PMC20574 9108111

[cne25157-bib-0013] Jarvis, E. D. , Güntürkün, O. , Bruce, L. , Csillag, A. , Karten, H. , Kuenzel, W. , et al. (2005). Avian brains and a new understanding of vertebrate brain evolution. Nature Reviews Neuroscience, 6(2), 151–159. 10.1038/nrn1606 15685220 PMC2507884

[cne25157-bib-0014] Jarvis, E. D. , Yu, J. , Rivas, M. V. , Horita, H. , Feenders, G. , Whitney, O. , et al. (2013). Global view of the functional molecular organization of the avian cerebrum: Mirror images and functional columns. The Journal of Comparative Neurology, 521(16), 3614–3665. 10.1002/cne.23404 23818122 PMC4145244

[cne25157-bib-0015] Karten, H. J. , Brzozowska‐Prechtl, A. , Lovell, P. V. , Tang, D. D. , Mello, C. V. , Wang, H. , & Mitra, P. P. (2013). Digital atlas of the zebra finch (*Taeniopygia guttata*) brain: A high‐resolution photo atlas. Journal of Comparative Neurology, 521(16), 3702–3715. 10.1002/cne.23443 PMC3931548 23896990

[cne25157-bib-0016] Kingsbury, M. A. , Kelly, A. M. , Schrock, S. E. , & Goodson, J. L. (2011). Mammal‐like organization of the avian midbrain central gray and a reappraisal of the intercollicular nucleus. PLoS One, 6(6), e20720. 10.1371/journal.pone.0020720 21694758 PMC3110203

[cne25157-bib-0017] Lein, E. S. , et al. (2007). Genome‐wide atlas of gene expression in the adult mouse brain. Nature, 445, 168–176. 10.1038/nature05453 17151600

[cne25157-bib-0018] Liu, H. , Liu, H. , Li, T. , Cui, J. , Fu, Y. , Ren, J. , et al. (2017). NR4A2 genetic variation and Parkinson's disease: Evidence from a systematic review and meta‐analysis. Neuroscience Letters, 650, 25–32. 10.1016/j.neulet.2017.01.062 28385514

[cne25157-bib-0021] Mello, C. V. , & Jarvis, E. D. (2008). Behavior‐dependent expression of inducible genes in vocal learning birds. In H. P. Ziegler & P. Marler (Eds.), The neuroscience of birdsong (pp. 381–397). Cambridge University Press.

[cne25157-bib-0022] Mello, C. V. , Kaser, T. , Buckner, A. A. , Wirthlin, M. , & Lovell, P. V. (2019). Molecular architecture of the zebra finch arcopallium. The Journal of Comparative Neurology, 516(3), 166. 10.1002/cne.24688 PMC6879308 30919954

[cne25157-bib-0023] Moon, M. , Jung, E. S. , Jeon, S. G. , Cha, M. Y. , Jang, Y. , Kim, W. , Lopes, C. , Mook‐Jung, I. , & Kim, K. S. (2019). Nurr1 (NR4A2) regulates Alzheimer's disease‐related pathogenesis and cognitive function in the 5XFAD mouse model. Aging Cell, 18(1), e12866. 10.1111/acel.12866 30515963 PMC6351845

[cne25157-bib-0024] Mouritsen, H. , Feenders, G. , Liedvogel, M. , Wada, K. , & Jarvis, E. D. (2005). Night‐vision brain area in migratory songbirds. Proceedings of the National Academy of Sciences United States of America, 102(23), 8339–8344. 10.1073/pnas.0409575102 PMC1149410 15928090

[cne25157-bib-0049] Pfenning, A. R. , Hara, E. , Whitney, O. , Rivas, M. V. , Wang, R. , Roulhac, P. L. , Howard, J. T. , Wirthlin, M. , Lovell, P. V. , Ganapathy, G. , Mountcastle, J. , Moseley, M. A. , Thompson, J. W. , Soderblom, E. J. , Iriki, A. , Kato, M. , Gilbert, M. T. P. , Zhang, G. , Bakken, T. , … Jarvis, E. D. (2014). Convergent transcriptional specializations in the brains of humans and song‐learning birds. Science, 346(6215), 1256846–1256846. 10.1126/science.1256846 25504733 PMC4385736

[cne25157-bib-0026] Puelles, L. (2014). Development and evolution of the claustrum. In The claustrum (pp. 119–176). Academic Press. 10.1016/B978-0-12-404566-8.00004-0

[cne25157-bib-0027] Puelles, L. (2017). Comments on the updated tetrapartite pallium model in the mouse and chick, featuring a homologous claustro‐insular complex. Brain, Behavior and Evolution, 90(2), 171–189. 10.1159/000479782 28988246

[cne25157-bib-0028] Puelles, L. , Kuwana, E. , Puelles, E. , Bulfone, A. , Shimamura, K. , Keleher, J. , Smiga, S. , & Rubenstein, J. L. R. (2000). Pallial and subpallial derivatives in the embryonic chick and mouse telencephalon, traced by the expression of the genes *Dlx‐2*, *Emx‐1*, *Nkx‐2.1*, *Pax‐6*, and *Tbr‐1* . Journal of Comparative Neurology, 424(3), 409–438. 10.1002/1096-9861(20000828)424:3<409::AID-CNE3>3.0.CO;2-7 10906711

[cne25157-bib-0029] Puelles, L. , Ayad, A. , Alonso, A. , Sandoval, J. E. , MartÍnez‐de‐la‐Torre, M. , Medina, L. , & Ferran, J. L. (2016). Selective early expression of the orphan nuclear receptor *Nr4a2* identifies the claustrum homolog in the avian mesopallium: Impact on sauropsidian/mammalian pallium comparisons. The Journal of Comparative Neurology, 524(3), 665–703. 10.1002/cne.23902 26400616

[cne25157-bib-0030] Reiner, A. , Perkel, D. J. , Mello, C. V. , & Jarvis, E. D. (2004). Songbirds and the revised avian brain nomenclature. Annals of the New York Academy of Sciences, 1016(1), 77–108. 10.1196/annals.1298.013 15313771 PMC2481519

[cne25157-bib-0032] Saha, R. N. , Wissink, E. M. , Bailey, E. R. , Zhao, M. , Fargo, D. C. , Hwang, J.‐Y. , et al. (2011). Rapid activity‐induced transcription of arc and other IEGs relies on poised RNA polymerase II. Nature Neuroscience, 14(7), 848–856. 10.1038/nn.2839 21623364 PMC3125443

[cne25157-bib-0033] Shimizu, T. , Bowers, A. N. , Budzynski, C. A. , Kahn, M. C. , & Bingman, V. P. (2004). What does a pigeon (*Columba livia*) brain look like during homing? Selective examination of ZENK expression. Behavioral Neuroscience, 118(4), 845–851. 10.1037/0735-7044.118.4.845 15301610

[cne25157-bib-0034] Smulders, T. V. , Sasson, A. D. , & Devoogd, T. J. (1995). Seasonal variation in hippocampal volume in a food‐storing bird, the black‐capped chickadee. Journal of Neurobiology, 27(1), 15–25. 10.1002/neu.480270103 7643072

[cne25157-bib-0036] Suzuki, I. K. , & Hirata, T. (2014). A common developmental plan for neocortical gene‐expressing neurons in the pallium of the domestic chicken *Gallus gallus domesticus* and the Chinese softshell turtle *Pelodiscus sinensis* . Frontiers in Neuroanatomy, 8(38), 783. 10.3389/fnana.2014.00020 PMC3985024 24778607

[cne25157-bib-0037] Thompson, C. L. , Ng, L. , Menon, V. , Martinez, S. , Lee, C.‐K. , Glattfelder, K. , et al. (2014). A high‐resolution spatiotemporal atlas of gene expression of the developing mouse brain. Neuron, 83(2), 309–323. 10.1016/j.neuron.2014.05.033 24952961 PMC4319559

[cne25157-bib-0050] Tokuoka, H. , Hatanaka, T. , Metzger, D. , & Ichinose, H. (2014). Nurr1 expression is regulated by voltage‐dependent calcium channels and calcineurin in cultured hippocampal neurons. Neuroscience Letters, 559, 50–55. 10.1016/j.neulet.2013.11.033 24291696

[cne25157-bib-0038] Volakakis, N. , Kadkhodaei, B. , Joodmardi, E. , Wallis, K. , Panman, L. , Silvaggi, J. , et al. (2010). NR4A orphan nuclear receptors as mediators of CREB‐dependent neuroprotection. Proceedings of the National Academy of Sciences United States of America, 107(27), 12317–12322. 10.1073/pnas.1007088107 PMC2901488 20566846

[cne25157-bib-0039] Wang, W. Z. , Oeschger, F. M. , Montiel, J. F. , García‐Moreno, F. , Hoerder‐Suabedissen, A. , Krubitzer, L. , et al. (2011). Comparative aspects of subplate zone studied with gene expression in sauropsids and mammals. Cerebral Cortex (New York, N.Y.: 1991), 21(10), 2187–2203. 10.1093/cercor/bhq278 21368089

[cne25157-bib-0040] Watakabe, A. , Ohsawa, S. , Ichinohe, N. , Rockland, K. S. , & Yamamori, T. (2014). Characterization of claustral neurons by comparative gene expression profiling and dye‐injection analyses. Frontiers in Systems Neuroscience, 8, 98. 10.3389/fnsys.2014.00098 24904319 PMC4033163

[cne25157-bib-0041] Watson, C. , & Puelles, L. (2017). Developmental gene expression in the mouse clarifies the organization of the claustrum and related endopiriform nuclei. The Journal of Comparative Neurology, 525(6), 1499–1508. 10.1002/cne.24034 27159785

[cne25157-bib-0042] Whitney, O. , Pfenning, A. R. , Howard, J. T. , Blatti, C. A. , Liu, F. , Ward, J. M. , et al. (2014). Core and region‐enriched networks of behaviorally regulated genes and the singing genome. Science, 346(6215), 1256780. 10.1126/science.1256780 25504732 PMC4359888

[cne25157-bib-0043] Wild, J. M. , Li, D. , & Eagleton, C. (1997). Projections of the dorsomedial nucleus of the intercollicular complex (DM) in relation to respiratory‐vocal nuclei in the brainstem of pigeon (*Columba livia*) and zebra finch (*Taeniopygia guttata*). The Journal of Comparative Neurology, 377(3), 392–413. 10.1002/(sici)1096-9861 8989654

[cne25157-bib-0044] Wullimann, M. F. (2017a). Should we redefine the classic lateral pallium? The Journal of Comparative Neurology, 525(6), 1509–1513. 10.1002/cne.24127 27670950

[cne25157-bib-0045] Wullimann, M. F. (2017b). Names matter: Commentary on Luis Puelles' article. Brain, Behavior and Evolution, 90(2), 190–190. 10.1159/000479784 28988242

[cne25157-bib-0046] Zapka, M. , Heyers, D. , Hein, C. M. , Engels, S. , Schneider, N.‐L. , Hans, J. , et al. (2009). Visual but not trigeminal mediation of magnetic compass information in a migratory bird. Nature, 461(7268), 1274–1277. 10.1038/nature08528 19865170

[cne25157-bib-0047] Zapka, M. , Heyers, D. , Liedvogel, M. , Jarvis, E. D. , & Mouritsen, H. (2010). Night‐time neuronal activation of cluster N in a day‐ and night‐migrating songbird. The European Journal of Neuroscience, 32(4), 619–624. 10.1111/j.1460-9568.2010.07311.x 20618826 PMC2924469

