# Supplementary figures and images for: Controlling for activity‐dependent genes and behavioral states is critical for determining brain relationships within and across species
Source: J Comp Neurol. 2021 May 4;529(12):3206–21. doi: 10.1002/cne.25157 (PMC8205984; doi:10.1002/cne.25157)

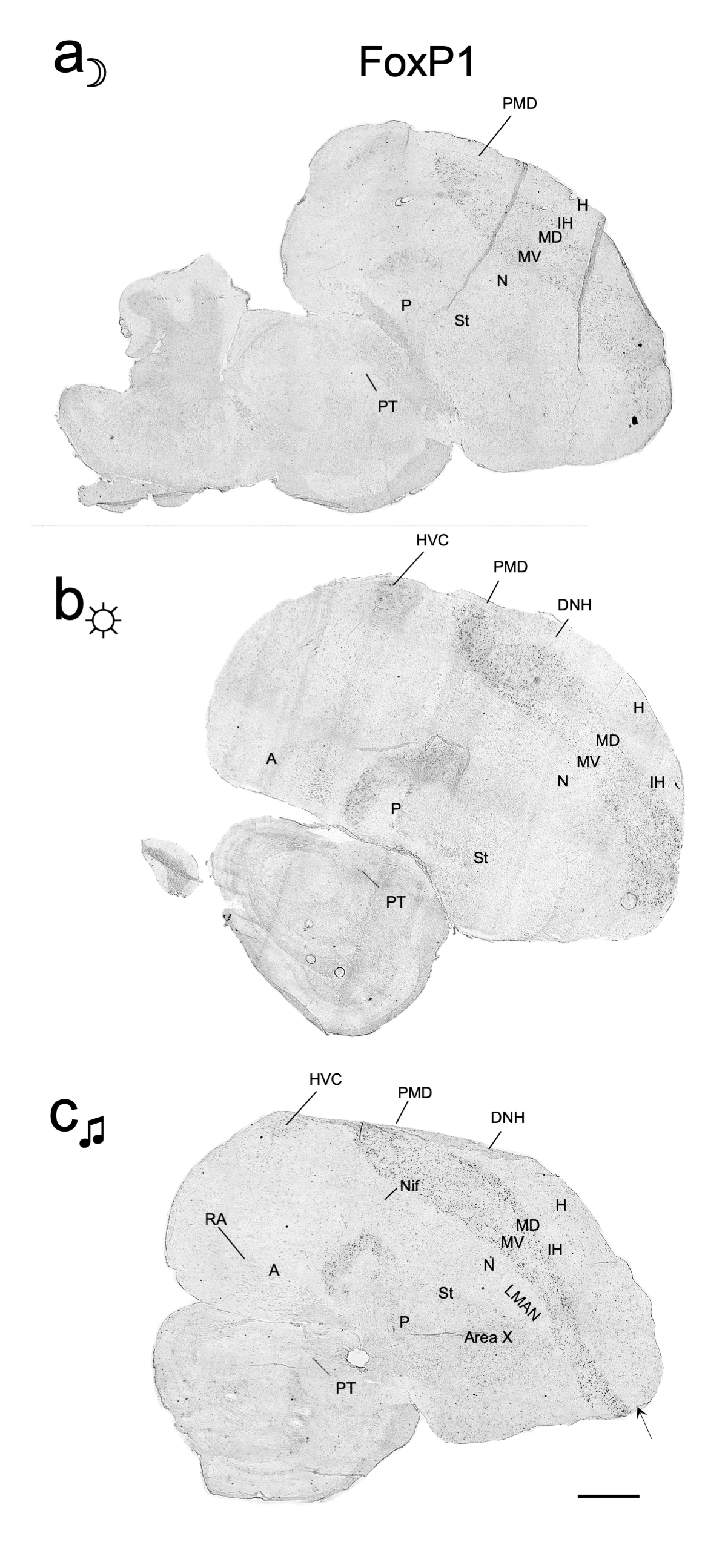

Supplement: Supplementary file 1 — FIGURE S1 Adjacent sections of the (a) dark silent, (b) light silent, and (c) singing animals in Figure 1, hybridized with FOXP1 to show the boundaries of the mesopallium with the hyperpallium and nidopallium. Tile imaged at ×4 magnification, scale bar = 1 mm. Dorsal is up, posterior is left. Abbreviations and corresponding names are shown in Table 1 [file CNE-529-3206-s001.tiff]
